# Supplementary material for: Mediation Effect of Obesity on the Association of Socioeconomic Status with Blood Pressure in the Elderly Hypertensive Population
Source: Nutrients. 2024 Jul 24;16(15):2401. doi: 10.3390/nu16152401 (PMC11314009; doi:10.3390/nu16152401)
Supplement: Supplementary file 1 [file nutrients-16-02401-s001.zip › nutrients-3065461-supplementary.pdf]

nutrients

MDPI

Supplementary Materials

Mediation Effect of Obesity on the Association of Socioeconomic Status with Blood Pressure in the Elderly Hypertensive Population

Supplementary Materials:

Table S1. The results of latent class models with different classes.
Table S2. Item-response probabilities in models with three latent classes.
Table S3. Characteristics of participants by socioeconomic status
Table S4. Demographic characteristics stratified by educational level.
Table S5. Demographic characteristics stratified by income.
Table S6. Demographic characteristics stratified by occupation.
Table S7. Mediation analysis of smoking and drinking on the association between socioeconomic status and BP control.
Figure S1. Flow chart of the study population selection.
Figure S2. Association of educational level, income, and occupation with BP control.

Table S1. The results of latent class models with different classes.

Classes	AIC	BIC	aBIC	Log likelihood	LMR	BLRT	Classification	Class probability
2	109845.680	109962.000	109914.331	-54907.840	0.000	0.000	8499/8735	0.49315/0.50685
3	109322.426	109500.782	109427.690	-54638.213	0.000	0.000	1127/8347/7760	0.06539/ 0.48433/0.45027
4	109322.792	109563.186	109464.669	-54630.396	0.056	0.064	10618/817/374/5425	0.61611/0.04741/0.02170/0.32225

Table S2. Item-response probabilities in models with three latent classes.

Item	Latent class 1	Latent class 2	Latent class 3
Education level			
Illiterate	0.119	0.385	0.569
Primary	0.268	0.376	0.292
Middle school	0.305	0.196	0.113
High school or above	0.309	0.043	0.027
Occupation			
Farmer	0.000	0.852	0.540
No-farmer	1.000	0.148	0.460
Income quartile			
Income quartile 1	0.025	0.070	0.492
Income quartile 2	0.121	0.198	0.350
Income quartile 3	0.154	0.379	0.157
Income quartile 4	0.699	0.352	0.000

Table S3. Characteristics of participants by socioeconomic status.

Variable	Low SES (N=7760)	Middle SES (N=8347)	High SES (N=1127)	*P*
**Gender, n (%)**				<0.001
Male	2603(33.5)	4072(48.8)	671(59.5)	
Female	5157(66.5)	4275(51.2)	456(40.5)	
**Age, years**	74.0(70.0,78.0)	72.0(68.0,76.0)	72.0(68.0,77.0)	<0.001
**Marital status, n (%)**				<0.001
Accompanied	4717(60.8)	6744(80.8)	932(82.7)	
Unaccompanied	3043(39.2)	1603(19.2)	195(17.3)	
**Drinking status, n (%)**				<0.001
Yes	6331(81.6)	5896(70.6)	771(68.4)	
No	1429(18.4)	2451(29.4)	356(31.6)	
**Smoking status, n (%)**				<0.001
Yes	7173(92.4)	7112(85.2)	895(79.4)	
No	587(7.6)	1235(14.8)	232(20.6)	
**Healthy physical activity, n (%)**				<0.001
Yes	5115(65.9)	6155(73.7)	802(71.2)	
No	2645(34.1)	2192(26.3)	325(28.8)	
**Major history diseases, n (%)**				
Diabetes	1890(24.4)	1918(23.0)	353(31.3)	<0.001
Stroke	2457(31.7)	2716(32.5)	307(27.2)	0.002
Coronary heart disease	1165(15.0)	1248(15.0)	124(11.0)	0.001
**Hypertension duration**	10.0(5.0,15.0)	10.0(5.0,15.0)	10.0(5.0,18.0)	<0.001
**Body mass index, kg/m²**	24.5(22.1,27.1)	24.6(22.4,27.01)	25.2(23.0,27.3)	<0.001
**Waist circumference, cm**	88.8(82.0,95.1)	89.0(82.0,95.1)	90.0(84.1,96.0)	<0.001
**SBP, mmHg**	142.0(130.1,154.7)	140.3(128.3,152.7)	136.3(125.7,148.7)	<0.001
**DBP, mmHg**	77.7(70.1,85.0)	77.5(70.5,84.7)	76.5(69.7,83.3)	0.001

Table S4. Demographic characteristics stratified by educational level.

Variable	Educational level			
	Illiterate	Primary	Middle school	High school or above
**Gender, n (%)**				
Male	1756(22.7)	2911(50.9)	2050(71.4)	629(68.8)
Female	5967(77.3)	2813(49.1)	823(28.6)	285(31.2)
**Age, years**	73.0(70.0,77.0)	74.0(70.0,77.0)	71.0(68.0,76.0)	69.0(66.0,74.0)
**Marital status, n (%)**				
Accompanied	5107(66.1)	4221(73.7)	2290(79.7)	775(84.8)
Unaccompanied	2616(33.9)	1503(26.3)	583(20.3)	139(15.2)
**Drinking status, n (%)**				
Yes	6757(87.5)	4063(71)	1624(56.5)	554(60.6)
No	966(12.5)	1661(29)	1249(43.5)	360(39.4)
**Smoking status, n (%)**				
Yes	7344(95.1)	4972(86.9)	2183(76.0)	681(74.5)
No	379(4.9)	752(13.1)	690(24.0)	233(25.5)
**Healthy physical activity, n (%)**				
Yes	6757(87.5)	4063(71)	1624(56.5)	554(60.6)
No	966(12.5)	1661(29)	1249(43.5)	360(39.4)
**Major history diseases, n (%)**				
Diabetes	1890(24.5)	1339(23.4)	678(23.6)	254(27.8)
Stroke	2461(31.9)	1850(32.3)	921(32.1)	248(27.1)
Coronary heart disease	1153(14.9)	870(15.2)	394(13.7)	120(13.1)
**Hypertension duration**	10.0(5.0,15.0)	10.0(5.0,15.0)	10.0(5.0,15.0)	10.0(5.0,16.0)
**Body mass index, kg/m²**	24.5(22.1,27.1)	24.6(22.3,27.0)	24.8(22.6,27.1)	24.8(22.6,27.2)
**waist circumference, cm**	88.0(81.2,95.0)	89.3(82.5,96.0)	90.0(83.0,96.0)	90.0(84.0,95.8)
**SBP, mmHg**	142.0(130.0,155.0)	140.5(128.7,152.7)	139.0(127.3,151.7)	137.7(126.6,150.7)
**DBP, mmHg**	77.3(70.3,84.7)	77.0(70.0,84.0)	78.3(71.5,85.3)	78.6(71.3,85.0)

Table S5. Demographic characteristics stratified by income.

Variable	Income			
	Income quartile 1	Income quartile 2	Income quartile 3	Income quartile 4
**Gender, n (%)**				
Male	1567(36.0)	1915(42.7)	2154(46.8)	1710(45.0)
Female	2788(64.0)	2566(57.3)	2448(53.2)	2086(55.0)
**Age, years**	74.0(70.0,78.0)	73.0(69.0,77.0)	72.0(68.0,76.0)	72.0(68.0,76.0)
**Marital status, n (%)**				
Accompanied	2053(47.1)	3626(80.9)	3755(81.6)	2959(78.0)
Unaccompanied	2302(52.9)	855(19.1)	847(18.4)	837(22.0)
**Drinking status, n (%)**				
Yes	4021(92.3)	3963(88.4)	3953(85.9)	3243(85.4)
No	334(7.7)	518(11.6)	649(14.1)	553(14.6)
**Smoking status, n (%)**				
Yes	3524(80.9)	3351(74.8)	3303(71.8)	2820(74.3)
No	831(19.1)	1130(25.2)	1299(28.2)	976(25.7)
**Healthy physical activity, n (%)**				
Yes	2801(64.3)	3084(68.8)	3417(74.3)	2770(73.0)
No	1554(35.7)	1397(31.2)	1185(25.7)	1026(27.0)
**Major history diseases, n (%)**				
Diabetes	1011(23.2)	1093(24.4)	1037(22.5)	1020(26.9)
Stroke	1313(30.1)	1418(31.6)	1449(31.5)	1300(34.2)
Coronary heart disease	627(14.4)	684(15.3)	652(14.2)	574(15.1)
**Hypertension duration**	10.0(5.0,15.0)	10.0(5.0,15.0)	10.0(5.0,15.0)	10.0(5.0,15.0)
**Body mass index, kg/m^2**	24.4(22.0,26.9)	24.6(22.3,27.1)	24.6(22.4,27.1)	24.7(22.5,27.1)
**waist circumference, cm**	89.0(82.0,95.0)	89.0(82.0,95.0)	89.0(82.0,95.0)	90.0(83.0,96.0)
**SBP, mmHg**	141.7(130.0,154.3)	141.0(129.0,153.7)	141.0(129.3,153.0)	140.0(127.5,152.3)
**DBP, mmHg**	77.3(70.0,84.7)	77.5(70.4,84.7)	78.0(71.0,85.0)	77.0(70.3,83.7)

Table S6. Demographic characteristics stratified by occupation.

Variable	Occupation	
	Famer	No-famer
**Gender, n (%)**		
Male	5057(44.5)	2289(39.0)
Female	6308(55.5)	3580(61.0)
**Age, years**	72.0(69.0,76.0)	74.0(70.0,78.0)
**Marital status, n (%)**		
Accompanied	8471(74.5)	3922(66.8)
Unaccompanied	2894(25.5)	1947(33.2)
**Drinking status, n (%)**		
Yes	9979(87.8)	5201(88.6)
No	1386(12.2)	668(11.4)
**Smoking status, n (%)**		
Yes	8416(74.1)	4582(78.1)
No	2949(25.9)	1287(21.9)
**Healthy physical activity, n (%)**		
Yes	3333(29.3)	1829(31.2)
No	8032(70.7)	4040(68.8)
**Major history diseases, n (%)**		
Diabetes	2615(23.0)	1546(26.3)
Stroke	3554(31.3)	1926(32.8)
Coronary heart disease	1644(14.5)	893(15.2)
**Hypertension duration**	10.0(5.0,15.0)	10.0(5.0,16.0)
**Body mass index, kg/m^2**	24.5(22.2,27.0)	24.7(22.4,27.2)
**waist circumference, cm**	89.0(82.0,95.0)	89.1(83.0,95.5)
**SBP, mmHg**	141.0(129.0,153.0)	141.0(129.0,154.0)
**DBP, mmHg**	77.3(70.0,84.5)	77.7(70.7,84.7)

Table S7. Mediation analysis of smoking and drinking on the association between socioeconomic status and BP control.

Mediating effect path (compared with low SES)	*β*	BootLLCI	BootULCI	*P*	Effect
**Middle SES—BP control (mediating variable: smoking)**					No mediating effect
Total effects	0.007	-0.008	0.021	0.360	
Indirect effects: Middle SES—smoking—BP control	-0.001	-0.003	0.001	0.280	
Direct effects: Middle SES—BP control	0.008	-0.008	0.022	0.280	
**High SES—BP control (mediating variable: smoking)**					No mediating effect
Total effects	0.086	0.050	0.107	<0.001	
Indirect effects: High SES—smoking—BP control	-0.001	-0.002	0.001	0.420	
Direct effects: High SES—BP control	0.086	0.051	0.107	<0.001	
**Middle SES——BP control (mediating variable: drinking)**					No mediating effect
Total effects	0.001	-0.002	0.023	0.220	
Indirect effects: Middle SES—drinking—BP control	0.001	-0.000	0.003	0.060	
Direct effects: Middle SES—BP control	0.008	-0.004	0.022	0.320	
**High SES—BP control (mediating variable: drinking)**					No mediating effect
Total effects	0.088	0.061	0.114	<0.001	
Indirect effects: High SES—drinking—BP control	0.002	-0.001	0.006	0.200	
Direct effects: High SES—BP control	0.086	-0.060	0.110	0.001	

Notes: SES: socioeconomic status. BP: blood pressure. All analyses control for gender, age, marital status, body mass index, waist circumference. and healthy physical activity, use of antihypertensive medication, numbers of antihypertensive medications, duration of hypertension at all paths.

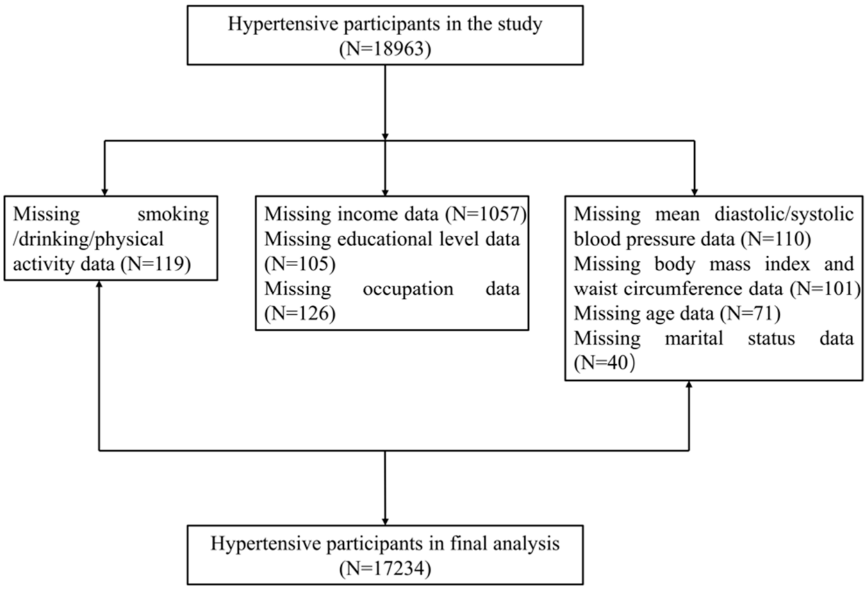

Figure S1. Flow chart of the study population selection.

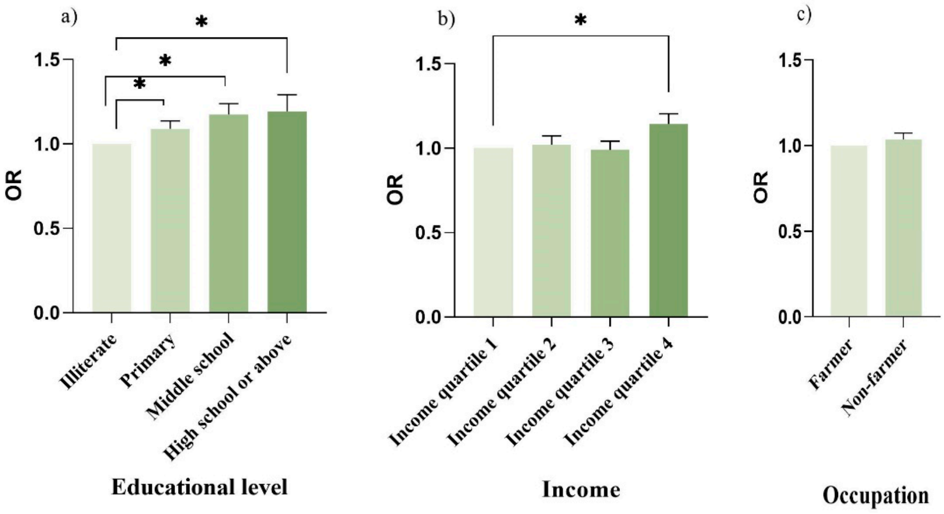

Figure S2. Association of educational level, income, and occupation with BP control.

Notes: (a): the association of educational level and BP control; (b): the association of income and BP control; (c): the association of occupation and BP control. The model adjusted for gender, age, smoking status, drinking status, healthy physical activity, marital status, and antihypertensive medication use situation, numbers of antihypertensive medications, hypertension duration. *: P<0.05.
